# Electrocardiographic recording in horses at rest: a concordance analysis of two methods

**DOI:** 10.1007/s11259-026-11486-9

**Published:** 2026-09-01

**Authors:** Isabel Latorre, Tomás Conde, Diana Marteles-Aragüés, Sergio Villanueva-Saz, María Teresa Verde, Antonio Fernández

**Affiliations:** 1 Veterinary Clinic “La Almunia-Cariñena”, La Almunia de Doña Godina, Zaragoza, 50100 Spain; 2 Department of Animal Pathology, Veterinary Faculty, Instituto Agroalimentario de Aragón-IA2 (Universidad de Zaragoza-CITA), University of Zaragoza, Zaragoza, 50013 Spain; 3 Clinical Immunology Laboratory, Veterinary Faculty, University of Zaragoza, Zaragoza, 50013 Spain

**Keywords:** Portable device, ECG, Horse, Arrhythmias, Concordance analysis

## Abstract

Cardiac arrhythmias are frequently detected during routine auscultation in athletic horses. Assessment of their clinical significance requires electrocardiography (ECG) to analyze waveforms and intervals. In recent years, portable ECG devices have been introduced to simplify electrocardiographic examination, offering advantages such as ease of use and the ability to review results via a smartphone. This study evaluated the utility and accuracy of a portable ECG device compared with a standard electrocardiograph. Simultaneous ECG recordings were obtained from 28 healthy adult horses at rest using a portable device (eKuore) and a standard electrocardiograph (Cardioline Vet600). Agreement between the two methods was assessed using Passing–Bablok regression and Bland–Altman analysis. All recordings were independently interpreted by two observers. A significant bias was observed between methods for heart rate, PR interval, and QRS duration, with higher values obtained using the portable device. Two arrhythmias were identified: second-degree atrioventricular block and atrial fibrillation. Agreement for arrhythmia detection was perfect (κ = 1.00), while agreement for heart rhythm classification (bradycardia, normal rhythm, or tachycardia) was good (κ = 0.73). In conclusion, the portable device demonstrated good diagnostic performance for arrhythmia detection during routine clinical examinations. However, further studies are required to establish reference intervals specific to this device.

## Introduction

During the physical examination of horses, assessment of cardiac function is an essential part of the diagnostic process, especially for detecting both physiological and pathological cardiac arrhythmias. A recent study by van Erck-Westergren et al. (2026) reported that 73% of pre-race electrocardiograms (ECGs) obtained from Thoroughbred racehorse, and 81% from poorly performing horses, exhibited at least one cardiac arrhythmia. Electrocardiography is the method of choice for accurately diagnosing arrhythmias in horses and other animals (Buhl et al. 2010; van Loon 2019; Decloedt et al. 2020; Alibrandi et al. 2024). However, in equine practice is not widely used, probably due to the low prevalence of heart disease in horses and the requirement for specialized equipment, including an electrocardiograph, cables, electrodes, and a power supply.

In small animal medicine, several methods have been evaluated for monitoring heart rate and detecting arrhythmias (Vezzosi et al. 2016; Alibrandi et al. 2024; Gunasekaran et al. 2025). These methods allow ECG tracings to be viewed on a smartphone or tablet and shared with other clinicians. In equine medicine, studies have also reported the use of smartphones for cardiac monitoring, typically placed on the animal’s left thorax (Vezzosi et al. 2018; Kraus et al. 2019; Corradini et al. 2020; Welch-Huston et al. 2020; Bindi et al. 2024). The recordings are usually exported as PDF files for interpretation; however, interpretation can sometimes be challenging because wave amplitudes and durations, as well as intervals, may differ from those obtained with a standard ECG.

This study aimed to compare the waveforms, amplitudes, and intervals of equine electrocardiograms (ECGs) recorded using a portable device with those obtained using a conventional electrocardiograph, and to assess the suitability of the portable device for ECG acquisition under field conditions.

## Materials and methods

### Animals

A total of 28 clinically healthy adult horses of various breeds were enrolled during pre-purchase examinations. The study population comprised both sexes, including 11 geldings, 1 stallion, and 16 mares. The median age was 14 years (range, 6–20 years). Horses were considered clinically healthy based on their medical history and physical examination, and all underwent electrocardiographic examination to screen for cardiac arrhythmias. During the pre-purchase examination, all horses underwent a routine physical examination, including cardiovascular assessment, to evaluate their general health. This evaluation consisted of respiratory and cardiac auscultation before and after exercise, a routine electrocardiographic examination, and a basic locomotor examination. Informed consent was obtained from all owners of subjects involved in the study. This protocol was approved by the Animal Experimentation Ethics Advisory Committee of the University of Zaragoza (Ref. PD36/20NE).

### ECG acquisition

The ECG recordings were performed in all horses by a single- lead, bipolar Ekuore ECG device and its smartphone application for Android (Chip Ideas Electronics SL, Valencia, Spain). All ECG tracings were acquired using a modified base-apex lead configuration, positioning the positive electrode attached in the right jugular groove near the thoracic inlet and the negative electrode attached on the left precordium (Vitale et al. 2021). This position was able to create a similar axis as a base apex lead which negative QRS complex deflection and positive P wave in the normal horse. An ECG recording was acquired for 30 s with a paper speed of 25 mm/s and an amplitude of 10 mm/mV for each horse. The application generates a PDF file for viewing on a smartphone. Simultaneously, an electrocardiogram, considered the gold standard, was performed using the Cardioline Vet600 electrocardiograph for 30 s. The yellow positive electrode was positioned on the left precordial area, the red negative electrode was positioned two-third to the way down the right jugular groove and the neutral electrode (black) was placed at the tip of the left shoulder. Lead II was selected to record the ECG. The paper speed was 25 mm/second, and an amplitude of 10 mm = 1 mv. The skin areas where the electrodes were placed were shaved and moistened with 70% alcohol to improve fixation and conductivity. To minimize artefacts, the electrodes were left in place until each horse had become accustomed to them, and only artefact-free recordings were included in the analysis. Only ECGs without artifacts were included in the study.

Two experienced observers independently evaluated the tracings obtained simultaneously using both methods in order to determine interval duration, wave polarity, and the presence of cardiac arrhythmias. The following parameters were recorded for both methods: heart rate beats/min (HR, bpm), P wave duration (s), PR interval (s), QRS complex (s), QT interval (s), P amplitude (mv) and R amplitude (mv). To calculate the HR per minute with both electrocardiographic recording methods, the QRS complexes were counted in 30 s and multiplied by 2. The automatic HR of the portable device was also recorded. For the duration (s) of each wave or interval, the average of three measurements was calculated, and the amplitude of the P and R waves (mv) was calculated using the average of three measurements for both electrocardiographic recording methods.

### Statistical analysis

Statistical analysis was conducted using MedCalc^®^ Statistical Software version 23.4.8 (MedCalc Software Ltd, Ostend, Belgium; https://www.medcalc.org; 2026). Concordance between the two electrocardiographic methods was evaluated using Passing-Bablok regression analysis, and the Bland-Altman method to provide limits of agreement between both methods. Lin’s concordance correlation coefficient (CCC) was used to assess the strength of the concordance between measurements. Cohen’s kappa concordance test was performed to calculate the agreement on the type of heart rate (bradycardia, normal, tachycardia), with bradycardia being less than 28 bpm, normal between 28 and 44 bpm and tachycardia if the rate was greater than 44 bpm (Reed et al. 2018). If the coefficient K was ≤ 0.20 was considered as no agreement; 0.21–0.40 as fair; 0.41–0.60 as moderate; 0.61–0.80 as good; 0.81–0.99 as very good, and 1 as perfect agreement. Statistical significance was defined as *p* ≤ 0.05.

## Results

**Fig. 1:**
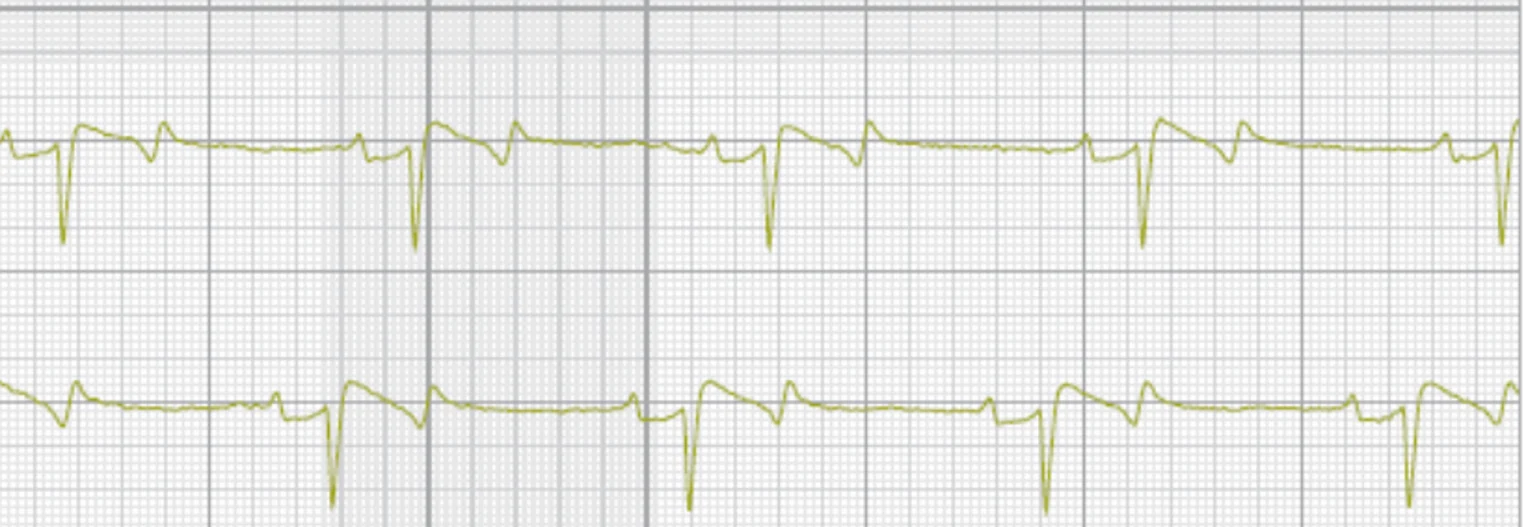
ECG obtained with the eKuore device at 25 mm/s, 10 mV from a mare. The ECG waves and intervals are clearly visible. Sinus rhythm, 40 beats per minute

This study compares a portable and a conventional method for ECG in horses. Fig. 1 shows the waves and intervals obtained with eKuore. The P wave showed a bifid positive polarity, the R wave a negative polarity, and the polarity of the T wave was variable, being able to be positive (11/28, 39.3%), or biphasic (17/28, 60.7%). Table 1 shows the Passing-Bablok and Bland-Altman results for both methods. A 2.9 bpm bias was found between the two methods for heart rate (HR), indicating a lack of agreement, with the HR for the eKuore device being higher than that of the conventional method. A large bias of 21.8 bpm was also observed between the HR automatically calculated by the portable device and the manually calculated HR, indicating poor agreement. A significant bias was also observed for the PR interval (-0.022 s), QRS complex (0.05 s), and P wave amplitude (-0.002 mv). Substantial agreement between both methods was observed for the P wave, QT interval and R amplitude. However, a constant bias inter device was found for QT interval (0.035 s), with the portable device reading being higher than the standard method, and also for R wave amplitude (0.01 mv). The correlation coefficients between the two methods were acceptable (*p* > 0.50) although poor (Lin’s rccc, *p* < 0.90), according to McBride (2005) criteria except for the P wave (Lin’s rccc 0.922).

The heart rhythm of 28 horses was sinus in 26 animals (92.8%), and two arrhythmias (7.1%) were detected with both methods: a second-degree atrioventricular block (1/28, 3.6%) and atrial fibrillation (1/28, 3.6%). Agreement between the two methods for distinguishing the presence or absence of arrhythmias was perfect (κ = 1.00). With the standard ECG, two horses were detected with bradycardia (7.1%, HR < 28 bpm) and seven with tachycardia (25%, HR > 44 bpm); with the eKuore device, one horse was detected with bradycardia (3.6%) and ten with tachycardia (35.7%). The agreement coefficient for heart rhythm types between the two methods was good (κ = 0.73; 95% CI 0.49–0.97).

**Table 1:** Analysis of concordance between methods of electrocadiographic registration. Intercept, slope and residual standard deviation of Passing-Bablok regression as well as bias, lower and upper limit of agreement from de Bland-Altman analysis and their 95% confidence intervals (95% CI) from the comparisons between the portable device and standard electrocardiographic. ¹HR (bpm): agreement between heart rate calculated manually from recordings obtained with the portable device and heart rate calculated from recordings obtained with the standard electrocardiograph. ²HR (bpm): agreement between the heart rate calculated automatically by the portable device and the heart rate calculated manually from recordings obtained with the portable device

Fraction		Passing-Bablok regression			Bland-Altman plot	
	Intercept (95% CI)	Slope (95% CI)	Residual standard deviation (95% CI)	Bias (95% CI)	Lower limit (95% CI)	Upper limit (95% CI)
^1^HR (bpm) ^2^HR (bpm)	-0.25 (-18 to 6) -34 (-107 to 3)	1.1 (0.9 to 1.5) 2 (1 to 3.8)	5.3 (-10.4 to 10.4) Cusum test = 0.25 28.4 (-56 to 56) Cusum test = 0.74	2.9 (0.03 to 5.8) 21.8 (-0.9 to 44.5)	-11.6 (-16.5 to -6.6) 93 (-132 to -54) -	17.3 (12.4 to 22.3) 136 (97 to 176)
P wave (s)	-0.003 (-0.017 to 0.04)	1 (0.29 to 1.14)	0.06 (-0.12 to 0.12) Cusum test = 0.96	-0.02 (-0.05 to 0.01)	-0.19 (-0.25 to 0.13)	0.14 (0.08 to 0.2)
PR interval (s)	0.03 (-0.04 to 0.08)	0.8 (0.67 to 1.09)	0.03 (-0.05 to 0.05) Cusum test = 0.82	-0.022 (-0.03 to -0.07)	-0.09 (-0.12 to -0.03)	0.05 (0.02 to 0.07)
QRS complex (s) QT interval (s)	-0.01 (-0.62 to 0.07) 0.035 (-0.18 to -0.12)	1.87 (0.86 to 5) 0.93 (0.67 to 1.22)	0.05 (-0.09 to 0.09) Cusum test = 0.58 0.06 (-0.12 to 0.12) Cusum test = 0.30	0.05 (0.02 to 0.07) 0.02 (-0.01 to 0.05)	-0.07 (-0.11 to -0.03) -0.15 (-0.21 to -0.09)	0.17 (0.13 to 0.21) 0.19 (0.13 to 0.25)
P amplitude (mv)	-0.09 (-0.3 to 0)	1.31 (1 to 2)	0.05 (-0.09 to 0.09) Cusum test = 0.99	-0.002 (-0.03 to -0.02)	-0.14 (-0.19 to -0.09)	0.14 (0.087 to -0.19)
R amplitude (mv)	0.01 (-5.2 to -0.2)	1.18 (1 to 1.6)	0.25 (-0.49 to 0.49)	-0.09 (-0.23 to 0.04)	-0.78 (-1.02 to -0.55)	0.6 (0.36 to 0.83)
			Cusum test = 0.58			

## Discussion

Arrhythmia detection is a fundamental component of the pre-purchase examination of competition horses. In recent years, portable devices have been developed in veterinary medicine to facilitate the diagnosis of cardiac disease in domestic animals (Kraus et al. 2019; Alibrandi et al. 2024). This study evaluated the agreement between ECG recordings obtained using the eKuore portable device and those obtained using a standard electrocardiograph (Cardioline Vet600). All waveforms recorded with the portable device were readily identifiable and showed morphology comparable to that obtained with the standard electrocardiograph. All electrocardiographic tracings were interpretable with both methods despite minor artefacts caused by movement or muscle tremor. The presence of artifacts is a problem when performing ECGs in horses, especially when using devices during exercise and not when they are at rest. These artefacts can be difficult to interpret in tracings where it is difficult to identify the P wave or the QRS complex. This is a common challenge encountered in other studies performing ECGs on horses (Welch-Huston et al. 2020; Vitale et al. 2021; ter Woort et al. 2022).

The heart rate (HR) calculated automatically by the eKuore device is sometimes very high and does not correspond to the actual value, which is calculated manually in the PDF file generated by the device, resulting in poor agreement. This is because the App algorithm uses the RR interval to calculate HR and sometimes interprets high T waves as beats, as if they were a RR interval. As indicated in other studies, it is not recommended to rely on the automatic HR generated by wireless devices in both horses (Kraus et al. 2019) and dogs (Vezzosi et al. 2016).

Many studies do not analyze the amplitude of ECG waves obtained with wireless devices (Kraus et al. 2019; Corradini et al. 2020; Welch-Huston et al. 2020) because they used devices with a small distance between the two electrodes (8–9 cm), resulting in low voltage and small waves that cannot be compared to those obtained with standard ECG. Brložnik et al. (2019) found that increasing the electrode distance to 50 cm increased the amplitude of the ECG waves. In our study, the electrodes were placed more than 50 cm apart, resulting in a P wave amplitude of 0.30 mV, the same as that reported by Brložnik et al. (2019). The results indicate good accuracy in the amplitudes of the P and R waves and suggest that the electrode placement and distance measured with the portable device were correct and the data are comparable to those obtained with the standard method. Furthermore, the wave polarity did not change, as in other studies (Kraus et al. 2019), and we obtained waves identical to those reported in other papers (Vitale et al. 2021; Spitale et al. 2024), but they did not evaluate the P and R amplitudes and so no comparison can be made. The best agreement between the two methods was observed for the P wave, QT interval, and R wave amplitude, where the bias found was not significant, according to the Bland-Altman analysis. Using the same wireless device in dogs, Alibandri et al. (2024) found good agreement for the duration of the intervals and, as in this study, perfect agreement in the detection of sinus rhythm, atrial fibrillation, and atrioventricular blocks. However, in our study, we did not find good agreement for the heart rate, as was the case in the study by Kraus et al. (2019) where wireless devices overestimated HR.

The agreement between the two devices for detecting cardiac arrhythmias was perfect in this case when evaluated in horses that did not exhibit signs of cardiac abnormalities. However, to better confirm the usefulness of the eKuore device for arrhythmia detection, it would be necessary to conduct the study in animals with pathological arrhythmias identified during the clinical examination of horses. In our work we only identified two arrhythmias, but Spitale et al. (2024) recorded up to six different types of cardiac arrhythmias with a larger population of horses in clinical practice with the eKuore device.

Although only two arrhythmias were detected, these results may indicate the usefulness of the eKuore device for diagnosing cardiac arrhythmias in field conditions, as it requires less handling and therefore causes less stress to the horse. Another advantage of portable devices is that, due to their ease of use and accuracy, they can be useful for monitoring chronic or recurrent cardiac arrhythmias by owners, as suggested by Kraus et al. (2019), similar to how it is done for dogs and in human medicine (Haberman et al. 2015), since the PDF file can be sent to a veterinarian for interpretation. The use of wireless devices for diagnosing cardiac arrhythmias is easy to perform and can be a useful diagnostic tool for the cardiological assessment of horses, especially in field conditions. The main limitation of this study was the small number of animals analyzed, and the need to evaluate it under field conditions with horses with arrhythmias detected by auscultation or in pre-purchase examinations of athletic horses, which are the horse population with the highest prevalence of cardiac arrhythmias (van Loon 2019; Decloedt et al. 2020; Nath et al. 2021; Kjeldsen et al. 2022; van Erck-Westergren et al. 2026).

In conclusion, the ECG recordings obtained with the portable device provided good diagnostic quality compared to the standard method. However, it is recommended to calculate the heart rate manually rather than using the automatic result. This device can be very helpful in field conditions for detecting cardiac arrhythmias during routine examinations of the horses; however, a limitation of the study was the small number of arrhythmias detected. Further studies are needed with more animals to establish reference intervals for the waveforms, intervals and amplitudes obtained in healthy horses with this portable device. 

## Data Availability

No datasets were generated or analysed during the current study.
